# Knowledge of COVID-19 among Health Care Workers at a Tertiary Care Hospital of Nepal: A Descriptive Cross-sectional Study

**DOI:** 10.31729/jnma.5266

**Published:** 2020-11-30

**Authors:** Sandip Bhandari, Medha Sharma, Gentle Sundar Shrestha

**Affiliations:** 1 Department of Anaesthesiology, Shahid Gangalal National Heart Centre, Kathmandu, Nepal; 2 Visible Impact, Kathmandu, Nepal; 3 Department of Anaesthesiology, Tribhuvan University Teaching Hospital, Maharajgunj, Kathmandu, Nepal

**Keywords:** *Corona-virus disease*, *health care workers*, *knowledge*

## Abstract

**Introduction:**

Health care workers are at higher risk of infection with the coronavirus disease as they are directly involved in the treatment of infected patients and perform aerosol-generating procedures. Proper knowledge of this disease can influence the positive attitude, good practices and enhance their safety. We aim to study the knowledge of COVID-19 among health care workers of the tertiary care hospital of Nepal.

**Methods:**

A descriptive cross-sectional study was conducted among health care workers of Shahid Gangalal National Heart Centre from May 20 to June 19, 2020. Ethical approval was taken from the Institutional Review Board (IRB No: 4-2020). Written informed consent was taken from all respondents. Correct answers were summated to reflect the mean knowledge, expressed as a percentage. Data analysis was done using Statistical Package for the Social Sciences version 21.

**Results:**

The mean general knowledge score was 95.7%. The mean medical knowledge score was 70.5%. Only 42 (56.8%) of physicians and 103 (53.6%) of nurses had a higher level of medical knowledge regarding COVID-19. Likewise, very few lab technicians 7 (21.9%) and none of the pharmacists had a higher level of medical knowledge.

**Conclusions:**

The healthcare workers of this centre have adequate knowledge regarding COVID-19. However, periodic training for all workers, especially the nurses and allied workers, may help to update the knowledge and hence enhance their safety and that of their patients.

## INTRODUCTION

Health care workers (HCWs) are at risk of Coronavirus disease (COVID-19) because of prolonged and repeated exposure to patients and because HCWs work in a team and physical distancing is usually not possible among them.^1^ HCWs of the cardiac centre are at even heightened as COVID-19 can manifest or precipitate as cardiovascular emergencies and also due to their involvement in aerosol-generating procedures (AGP).^2^

Poor knowledge and misunderstanding have been found to lead to delayed treatment,^3^ recognition, and handling of potential COVID-19 patients, ultimately leading to its rapid spread in hospitals.^4,5^

Thus, this study aims to investigate the knowledge of COVID-19 among HCWs of tertiary level cardiac care hospital in Nepal. This will help to generate evidence that can support the hospital management to design interventions to ensure the safety of HCWs and patients at this time of the pandemic.

## METHODS

This is a descriptive cross-sectional study conducted among HCWs of Shahid Gangalal National Heart Centre from May 20 to June 19, 2020. Ethical approval was taken from Institutional review board (IRB No: 4-2020), Shahid Gangalal National Heart Centre and written informed consent was taken from all respondents. Participants were enrolled using convenient sampling technique. The sample size was calculated using the formula,

$$\begin{array}{ll} \text{n} & \text{= }{\text{Z}}^{\text{2}}\times \text{p}\times \text{(1}-\text{p)}/{\text{e}}^{\text{2}}\\ & \text{= }{\left(1.96\right)}^{\text{2}}\times 0.5\times 0.5/{(\text{0.06)}}^{\text{2}}\\ & \text{= }267 \end{array}$$

Where,

n = sample size
Z = 1.96 at 95% Confidence Interval (CI)
p = population proportion, 50%
e = margin of error, 6%

Taking a 10% non-respondent rate, the sample size became 294. However, data was collected from 305 HCWs.

The data was collected through a self-administered questionnaire, consisting of three sections: socio-demography, general knowledge, and medical knowledge. Correct answers were summated to reflect the mean knowledge, expressed as a percentage. The socio-economic status was analyzed using 5 household items (mobile, house, laptop, vehicle, internet).^6^

Data analysis was done using Statistical Package for the Social Sciences (SPSS) version 21.

## RESULTS

The mean basic score was 95.7%. All general knowledge factors were answered correctly by more than 90% of respondents except for the knowledge related to disease symptoms, which was correctly answered by 219 (71.8%) of respondents only. General knowledge about COVID-19 was assessed using 10 factors (Table 1).

**Table 1:** General knowledge components of the COVID-19 questionnaire and the correct responses.

Questions		Correct responses n(%)
1.	The COVID-19 virus (SARS-CoV-2) spreads via respiratory droplets of infected individuals.	298 (97.7)
2.	There currently is no effective cure for COVID-19 but early symptomatic and supportive treatment can help with most patients recover from the infection.	302 (99)
3.	Health care workers are at higher risk of infection.	301 (98.7)
4.	The main clinical symptoms of COVID-19 are fever, fatigue, sore throat, dry cough and myalgia.	304 (99.7)
5.	To prevent the infection by COVID-19 individuals should avoid going to crowded places such as the gym, theatres and avoid public transportation.	303 (99.3)
6.	Isolation and treatment of people who are infected with the COVID-19 virus are effective ways to reduce the spread of the virus.	303 (99.3)
7.	Unlike the common cold, stuffy nose, runny nose, and sneezing are less common in persons infected with the COVID-19.	219 (71.8)
8.	A vaccine is now publicly available for COVID-19.	287 (94.1)
9.	Contact persons should be isolated for at least 14 days.	297 (97.4)
10.	Hand hygiene, covering nose and mouth while coughing, and avoiding sick contacts can help in the prevention of COVID-19 transmission.	305 (100)

The mean medical knowledge score was 70.5%. Dichotomizing the medical knowledge score through the median, only 56.8% of physicians and 53.6% of nurses had a higher level of medical knowledge regarding COVID-19. None of the pharmacists and only 21.9% of lab technicians/radiographers had a higher level of medical knowledge.

Regarding the N95 respirators, only 141 (46.2%) and 92 (30.2%) of HCWs respectively knew that they are not recommended for use in OPD and triage room respectively. However, 268 (87.9%) knew they should be used during tracheal intubation. Regarding PPE, 262 (85.9%), 268 (87.9%) and 254 (83.3%) of HCWs respectively knew that they are used in tracheal intubation, surgery of suspected case, and for collecting the respiratory sample. However, only 138 (45.2%) knew that they are not recommended for use in OPD of infectious disease. The importance of category I PPE donning during CPR for potential COVID-19 patients is known to 294 (96.4%) of HCWs. And 143 (46.9%) and 277 (90.8%) of HCWs respectively knew that patients should be intubated early and experts should do it. The recommendation for not giving rescue breathing is known by 235 (77%) HCWs. Regarding the infection reduction, 278 (91 %) and 253 (83%) of HCWs knew that COVID-19 infection can be reduced by separating COVID and non-COVID patients and staff education respectively. Only124 (40.7%) knew it can be reduced by decreased mobile use.

Only 167 (54.8%) of respondents knew about the correct use of surgical and N95 masks. Only 120 (39.3%) knew that these masks can be sterilized. A very high proportion 252 (82%) of HCWs knew alcohol can't and 248 (81.3%) knew that UV light can sterilize them (Table 2).

**Table 2:** Medical knowledge components of the COVID-19 questionnaire and the correct responses.

Knowledge Factors	Correct response n (%)
1. Antibiotics as the first line of treatment for COVID-19	251 (82.3)
2. Use of surgical mask for non-AGP	279 (91.5)
3. Persons with COVID-19 cannot infect the virus to others when the fever is not present?	291 (95.4)
4. Recommendation of use of N95 respirator	196 (64.3)
5. Requirement of PPE	230 (75.6)
6. Regarding the decrease of COVID-19 spread	201 (65.9)
7. CPR for potential COVID-19 patients	237 (77.8)
8. Regarding reuse and sterilization of surgical and N95 mask	167 (54.8)

The source of knowledge regarding COVID-19 was mostly social media (87%), while 62%, 60%, and 56% of respondents referred to digital media, national TV/ radio, and friends or relatives to obtain such information respectively. Interestingly, only 2.29% of HCWs referred to more authentic sources such as classes, webinars, and journals for information.

Out of 305 HCWs, 192 (63%) were nurses, 75 (24.5%) physicians, 32 (10.5%) lab technicians/radiographer, and 6 (2%) pharmacists. The mean age of the respondents was 31.2 + 6.7 years, (range; 20-59 years). Approximately 55% of the respondents were aged 20-30 years. Most (74.1%) were female. Most (60.3%) respondents were married/living together. Ethnic groups were almost equally represented (Janajati = 37.4%, Brahmin = 31.1%, Chhetri = 31.1%). The mean economic status was 68%. Approximately 44.3%, 54.7% and 2% of HCWs were on higher, moderate and lower economic category respectively. The mean years of professional experience were 8.3 + 6.9 years (range; 1-36 years).

## DISCUSSION

COVID-19 has emerged as a significant public health concern, especially after the World Health Organisation (WHO) declared it as a pandemic on 11^th^ March 2020.^7^ Nepal had its first COVID-19 case on January 24, 2020. As of 13 July 2020, Nepal has 17,658 cases with 40 mortalities. Till date, 85 HCWs have been infected with COVID-19 but no mortality has been reported yet.^8^

Patients of cardiovascular comorbidity are at higher risk of contracting COVID-19 and have a worse prognosis with a case fatality rate of 10.5% which is higher than the average population.^2^ COVID-19 patient may present as a direct manifestation of or precipitation of previous cardiac symptoms. Their classic symptoms and presentation may be overshadowed leading to under-diagnosis.^2^ When patients present with cardiovascular emergencies like acute myocardial infarction, arrhythmias, heart failure, etc. There is no time to confirm or rule out COVID-19and HCWs in such settings might need to perform AGP such as tracheal intubation, cardiopulmonary resuscitation (CPR), respiratory sample collection, etc.^2^ Due to prolonged and repeated exposure to patients, and the job nature of working in a team, physical distancing is usually not possible among HCWs.^1^ Likewise, limited knowledge of the disease itself, limited availability and improper donning and doffing of personal protective equipment (PPE), etc. might increase the risks of COVID-19 infection.^1^ Hence, HCWs working in cardiac centre are at heightened risk of COVID-19 infection.

As no definitive antiviral treatment or vaccine has been explicitly recommended for COVID-19, exercising preventive measures to control its spread is the only most widely accepted intervention recommended.9 As HCWs are involved in the treatment of COVID-19 patients, their knowledge of prevention of disease transmission becomes must so that they can prevent COVID-19 infection by decreasing the risk of transmission, recognizing symptoms early, and assisting in diagnosis. When this disease became pandemic earlier this year, online courses for awareness of HCWs were initiated10 regarding COVID-19. However, the level of knowledge of this disease among HCWs in Nepal is still largely unknown. Thus, this research aimed to investigate knowledge of HCWs about the COVID-19 in a national cardiac care centre of Nepal.

The mean general knowledge score in this study (95.7%) was slightly higher than the study by Huynh et al.^11^ and Zhang et al.^12^ where 88.4% HCWs and 89% HCWs demonstrated sufficient knowledge of COVID-19. Also, knowledge score of preventive measures such as avoiding crowded places like gym, theatres, and public transportation, etc. isolation of people infected with COVID-19 as effective measures to reduce the spread of the virus are quite higher compared to similar studies. In the study done by Huynh et al, the contact person should be in isolation for at least 14 days is known by 65.8% HCWs only^11^ which is known by 97.4% HCWs in our study. Similarly, regarding knowledge of transmission, covering nose and mouth while coughing and avoiding symptomatic contact can help prevent COVID-19 transmission is known by 100% HCWs and 97.7% HCWs knew that it is mainly transmitted by droplets. These results are better than the study done by Modi et al, where 62% responders knew the main mode of transmission is via respiratory droplets.^13^

This higher knowledge in this study might be attributed to the fact that this study was done a few months later than previous studies, and HCW might have been able to obtain more information about the disease from Infection Prevention and Control Committee of this centre, and other sources such as Ministry of Health and Population (Nepal), WHO and Center for disease control and prevention.

Till date, Remdesivir^9^ and Dexamethasone^14^ have some evidence and evidence is emerging for Tocilizumab^15,16^ for COVID-19 treatment. The vaccine is not available for COVID-19 is known by 89.3% HCWS in the study by Huynh which is very similar to our study where 94.1% HCWs knew about it.^11^

The basic symptoms of COVID-19-fever, fatigue, sore throat, dry cough etc. was only known to 84% physicians, 69% nurses, 66.7% pharmacists and 56.2% lab technicians/radiographers. This is very similar to the study done by Huynh^11^ where symptoms of COVID-19 were known by 72.8% of HCW.

AGP requires the use of N95 respirator or equivalent.^17^ Approximately, 91.5% of HCWs believe that they can wear a surgical mask for non-AGP. Knowledge for N95 respirator was 64.1% but 87.9% of HCWs knew that the N95 mask is recommended for tracheal intubation. At this time, when these masks are running short in supplies, it becomes important to use the N95 mask appropriately. Along with this, appropriate techniques of its use, removal, storage between use and reuse if not visibly soiled should be known to all HCWs.^18^ In this study, 46.2% and 30.2% of HCWs respectively don't think N95 should be used in OPD and triage room. Only 39.3% of HCWs know that it can be reused.

Awareness of proper use of PPE is known to 75.6% of HCWs and on average 85.7% of HCWs feel they should use it while intubating, performing surgery on the suspected patient and while collecting the respiratory sample. WHO has made guidelines for proper use of PPE to ensure there is no undersupply of PPE at the time of real need.^19^ Before caring for patients with confirmed or suspected COVID-19, HCWs must receive comprehensive training on why and when is PPE necessary, how to don and doff PPE, as well as proper care, maintenance, and disposal of PPE. HCWs also need to demonstrate competency in performing appropriate infection control practices and procedures, donning and doffing in sequence, and they should not adjust PPE (e.g., retying gown, adjusting respirator/facemask) during patient care to prevent self-contamination.^1^

Because there is no vaccine developed till date and that 82.3% are aware that antibiotics are not the treatment of choice, it can be inferred that HCWs have no other option but to maintain the utmost precautions while treating the patients. Basic measures like separating COVID and non-COVID patients, installing a barrier to limit contact with the patient at triage, prioritizing respirators for AGP, self-monitoring sign of illness, self-isolating, reporting any illness etc. are required for better case management and prevention of the spread of COVID-19.^1,20^

CPR being one of the high-risk procedure to transmit COVID-19 infection, there have been some changes in the American Heart Association and European Resuscitation Guideline for CPR of suspected or known COVID-19 patients.^21^ The study showed 77.8% of HCWs know CPR of such patients referring that a good percent of HCWs of this center has already updated themselves on those CPR guidelines.

This study revealed that the majority (87%) of HCWs gather their information through social media-Facebook, twitter etc. Interestingly, only 2.29% of HCWs referred to more authentic sources like classes, webinars, journals etc. for information. Since the knowledge of respondents in this study is quite high, they referring to social media for information shows that the use of social media is increasing and gaining trust globally. However, the government should be vigilant and scrutinize the use of social media ensuring only authentic and updated information about COVID-19 is disseminated. Also, the fact that decreased use of gadgets like mobiles can also decrease the transmission is known to only 65.9% HCWs, and their increased use of social media might increase the risk of disease transmission through these gadgets as it might act as fomites.

On average, the overall knowledge of the physicians and nurses is better than allied HCWs. However, only around half of the physicians and nurses had a higher level of medical knowledge. This highlights the need to impart further knowledge to the HCWs.

There are various limitations to this study. Firstly, this study was conducted among the HCWs in the cardiac centre of an urban area with good access to modern teaching-learning and internet facilities-webinar. So, the result might not be generalized to other multidisciplinary and rural centres. Secondly, only the knowledge was assessed and not skills. Knowledge is also obtained hands-on, meaning HCWs might be learning things while they practice. The 100% knowledge of HCWs regarding hand hygiene might be due to pre-existing proper hand hygiene practices in the center of study.^22^ Conversely, the HCWs might have theoretical knowledge about the proper use of PPE, but may not be able to apply in practice (eg. unable to doff). Not being able to measure skill limits the study in being able to triangulate the knowledge and skills. The study was conducted among the HCWs only while knowledge of non-HCWs about COVID-19 is also important to prevent its transmission.

## CONCLUSIONS

HCWs from this centre have a better knowledge of COVID-19 than previous studies, though it differs significantly among physicians, nurses, and allied HCWs. As the understanding of this disease is rapidly evolving, there is a constant need for updating the HCWs regarding new protocols and guidelines for COVID-19. Also, with a shortage of PPE, the knowledge of use/reuse of appropriate PPE becomes very important. As there is a possibility of long-term existence of SAR-CoV-2 but no definite treatment till now, COVID-19 might become an even bigger public health emergency in the future. This has increased the need to provide ongoing training on the use of appropriate PPE with the appropriate technique of donning and doffing and additional education and training to manage the surge if it occurs. Therefore, this study recommends the hospital management to arrange for periodic webinars, online modules, or other sources of authentic information for all HCWs, with special focus on allied HCWs.
